# Consequences of drive on the evolution of genomes and species

**DOI:** 10.1093/g3journal/jkaf076

**Published:** 2025-04-24

**Authors:** Logan T Edvalson, Amanda M Larracuente

**Affiliations:** Department of Biology, University of Rochester, Rochester, NY 14627, United States; Department of Biology, University of Rochester, Rochester, NY 14627, United States

**Keywords:** meiotic drive, hybrid incompatibilities, arms race

## Abstract

Meiotic drivers violate Mendelian principles by biasing their segregation to the next generation, often at a cost to the individual. This cost leads to a cyclical arms race between drivers and host genomes where drivers acquire modifications to cheat, and the rest of the genome evolves counter-drive mechanisms to restore fair segregation. Repeated bouts of innovation on both sides of the conflict can have consequences for genome and species evolution. Much of the foundational and recent work in this field has found its home in the Genetics Society of America journals. In this commentary, Edvalson and Larracuente highlight some recent work published in these journals that address key questions about the evolutionary consequences of meiotic drive.

## Meiotic drive shapes genome evolution

Meiosis ordinarily ensures the fair segregation of alleles to gametes, a fundamental principle of Mendelian genetics. Meiotic drivers (hereafter drivers) are selfish genetic elements that subvert the fairness of meiosis by biasing their transmission to the next generation at the expense of the other allele. Drivers deploy diverse strategies to cheat their way into the next generation. Some cheat by killing rival gametes like the X-linked sperm killers that distort sex ratios in *Drosophila* species. Others cheat by preferentially segregating to functional gametes, like the selfish “*D*” centromeres in *Mimulus guttatus* (Lindholm *et al*. 2016). Drive is widespread across insects, fungi, plants, and mammals and can have large effects on genome evolution (Lindholm *et al*. 2016).

Drivers often impose costs on their bearers—either directly due to the drive mechanism itself or through the accumulation of linked deleterious mutations. The transmission advantage enjoyed by the driver allows it to spread in populations even when it is costly, creating selection pressure for hosts to evolve drive suppression. Drivers and their hosts can thus get locked into evolutionary arms races where cyclic rounds of drive and suppression result in rapid evolution (reviewed in Lindholm *et al*. 2016). This rapid evolution can have consequences. Below, we discuss some recent papers published in GENETICS and G3 that provide new insights into some drive consequences and their implications for genome and species evolution.

## New insights into drive and hybrid incompatibilities

The rapid evolution triggered by drive-related arms races can contribute to reproductive isolation between populations with consequences for interspecies hybrids. The consequences can be indirect—when the rapid divergence of drive system components incidentally lead to sperm dysfunction—or direct, when male sterility results from drive itself. When suppressors of drive reach fixation in a population, the driver loses its advantage and becomes cryptic. Populations evolving in isolation can accumulate different driver–suppressor combinations. When these populations come into contact, cryptic drivers unleashed in hybrids may cause male sterility (reviewed in Meiklejohn and Tao 2010). The role of drive in the process of speciation is a debated topic in genetics and evolution. One concrete connection between drive and incompatibilities occurs in hybrids between *Drosophila pseudoobscura* subspecies—one from the United States (USA) and one from Bogota, Colombia (Bogota). In interspecific crosses where Bogota is the mother, both hybrid sterility and drive-related sex ratio distortion map to a single locus on the Bogota X chromosome called *Overdrive* (Phadnis and Orr 2009).

Recent work from Bladen *et al*. in GENETICS may provide an additional connection between drive and hybrid incompatibilities in crosses between the same subspecies of *D. pseudoobscura*, but in the opposite direction (Bladen *et al*. 2024). USA populations have a well-studied X-linked meiotic drive system called *Sex Ratio* (*SR*). Genetic crosses between USA mothers and Bogota fathers generally result in fertile F1 hybrid offspring. If the mother carries an *SR* chromosome, then her hybrid sons will exhibit sex ratio distortion (Bladen *et al*. 2024) suggesting that the Bogota genome does not have any (dominant) suppressors of drive.

However, Bladen *et al*. discovered that introgressing the *SR* chromosome into a homozygous Bogota background produces mostly sterile sons, whereas the same crosses with a standard nondriving X chromosome produces fertile sons (Bladen *et al*. 2024). These authors may have discovered another connection between drive and hybrid sterility. In hybrids, *SR* might “misfire” and indiscriminately kill all gametes, including those with the driver. Although there are no known current suppressors of *SR* in USA, they propose that this species has experienced historical waves of drive and suppression. At present, *SR* may have the edge. Bogota might be missing a suppressor that evolved in a previous cycle of the USA driver–suppressor arms race (Bladen *et al*. 2024). This study provides new insights into drive system evolution between closely related subspecies. The genetic basis of both drive and sterility awaits further characterization: *SR* is a complex multi-locus system with chromosomal inversions. If a direct link between *SR* and hybrid sterility is confirmed by future experiments, it may present a great model system to study drive misfire.

Divergence in drive systems can also create incompatibilities *within* species. In the fission yeast *Schizosaccharomyces pombe*, crosses between isolates can be sterile due to mismatched drivers (e.g. see López Hernández *et al*. 2024). The *with tf* (*wtf*) drivers in *S. pombe* are part of a rapidly evolving multi-copy gene family. Each driver encodes both a poison and an antidote. Spores without an antidote are destroyed by the poison, and some antidote-only copies act as suppressors. Because poison-containing drivers must have a matching antidote, divergence between *wtf* drivers contributes to inter-isolate incompatibilities. *Wtf* genes have duplicated and spread within *S. pombe* such that isolates contain a variable number of distinct *wtf* loci. It is unclear why this species harbors so many polymorphic drivers.

A recent G3 paper from López Hernández *et al*. provides some new insights into the evolution of driving multi-copy gene families. They model the evolutionary dynamics of duplicate drivers (López Hernández *et al*. 2024). When discussing the arms race caused by genetic conflicts, we typically envision the conflict between the selfish element (e.g. the driver) and the genome. Here, the authors capture conflicts *between drivers* and show that there can be selection pressure for a driver to diverge even in the absence of suppressors (López Hernández *et al*. 2024). Distinct drive loci may spread under a wide range of conditions. Their model might help explain how multiple drivers can arise, diverge, and spread in populations.

## Detecting drive and its consequences

Drivers are transient in populations. Depending on the stage of the arms race, they can be difficult to detect. Understanding the prevalence of drive and the extent of its impact on genome and species evolution is an ongoing challenge. The “classic” drive systems generally have large phenotypic effects and were discovered serendipitously in genetic studies. While difficult to detect in the lab, weak drive can have impacts over evolutionary timescales. [See the recent GENETICS paper suggesting that weak female drive might shape patterns of inversion polymorphisms in *Drosophila melanogaster* (Koury 2023) as an example.] Large-scale screens of gametes or pooled individuals (e.g. Wei *et al*. 2017) can help identify weak signatures of drive for further exploration (Barbash *et al*. 2023). As methods for detecting and confirming drive improve, future surveys may help shed light on how abundant drivers are in nature.

We are still learning about the diverse mechanisms that drivers use to bias their transmission. Understanding how drive affects genetic, cellular, and molecular processes may reveal important information about how gametogenesis is vulnerable to cheaters. Genetic and genomic approaches are powerful for dissecting drive mechanisms and can help distinguish between the drive of selfish genetic elements and other types of non-Mendelian segregation. Recent studies of chromosomes with unusual inheritance patterns with parallels to selfish driving chromosomes, like the *Social* supergene (*Sb*) in the fire ant, *Solenopsis invicta* (Hettesheimer *et al*. 2025), and nullo-X spermatid elimination in the nematode, *Auanema freiburgense* (Al-Yazeedi *et al*. 2024), may provide insights into different ways that chromosomes can achieve biased transmission.

Advances in genomics and genome editing technology expand the reach of this field into nontraditional model systems and practical applications. The lessons learned from studying natural drive systems may have implications for addressing large-scale global problems. Experimental synthetic drive systems modeled after natural drivers can be used to manipulate populations: e.g. control vectors of human disease or provide resistance to disease and pests. GENETICS and G3 have been homes for recent papers on synthetic drive and its applications (e.g. Terradas *et al*. 2022; Lawler *et al*. 2024).

Several outstanding questions remain at the center of this field: What are the molecular mechanisms of drive? How prevalent is drive in nature and what is the extent of its influence on genome and species evolution? How can theoretical and empirical studies of drive systems help build safe solutions to pressing global challenges? Progress here requires interdisciplinary approaches and diverse systems. Whether the approach involves molecular genetics or theoretical and population genomics, natural or synthetic systems, and traditional or emerging model organisms, GENETICS and G3 will continue to be a place for intercommunity discourse on this fascinating topic.
